# Gender, ethnicity, health behaviour & self-rated health in Singapore

**DOI:** 10.1186/1471-2458-7-184

**Published:** 2007-07-27

**Authors:** Wei-Yen Lim, Stefan Ma, Derrick Heng, Vineta Bhalla, Suok Kai Chew

**Affiliations:** 1 Epidemiology & Disease Control Division, Ministry of Health Singapore, 16 College Road S(169854)

## Abstract

**Background:**

Self-rated health and the factors that influence it have never been described in Singapore before. This paper presents a descriptive study of self-rated health in a nationally representative cross-sectional survey of 6236 persons.

**Methods:**

As part of the National Health Surveillance Survey 2001, 6236 subjects aged 18 years and above were interviewed in the homes of participants by trained interviewers. The subjects were asked "In general, how would you rate your health today?", and given 5 possible responses. These were then categorized as "Good" (very good and good) and "Poor" (moderate, bad and very bad) self-rated health. The association of socio-economic and health behaviour risk factors with good self-rated health was studied using univariate and multivariate logistic regression analysis.

**Results:**

Univariate analyses suggest that gender, ethnicity, marital status, education, household income, age, self-reported doctor-diagnosed illnesses, alcohol intake, exercise and BMI are all associated with poor self-rated health. In multivariate regression analyses, gender, ethnicity, household income, age, self-reported illness and current smoking and BMI were associated with poor self-rated health. There are gender differences in the association of various factors such as household income, smoking and BMI to self-rated health.

**Conclusion:**

Socioeconomic factors and health behaviours are significantly associated with self-rated health, and gender differences are striking. We discuss why these factors may impact self-rated health and why gender differences may have been observed, propose directions for further research and comment on the public policy implications of our findings.

## Background

Self-rated health is easily measured in large population surveys, and is a useful "opener" in interview situations that allow interviewers to seek more nuanced and complex responses about people's perceptions of their health.

Multiple studies, conducted in a variety of cultures and settings, have consistently shown that persons reporting poorer self-rated health suffer a higher subsequent risk of mortality. Such studies have spanned a wide range of populations, from persons with illnesses such as cancer [1] and cardiovascular disease [2], to the elderly [3-5], and to general populations [6-8]. Poor self-rated health has also been shown to be independently predictive of subsequent morbidity and higher healthcare utilization [9,10].

The consistent association between self-rated health and subsequent adverse outcomes may be due to a number of factors: people evaluate self-rated health holistically, taking into account a variety of social, physical and emotional factors that impact well-being; people may include evaluations of vague symptoms or disease in the preclinical stage in assessing overall health; they may consider the past trajectory of their physical and mental functioning and their expectation of future functioning; or they may consider the resources available to them, both in the present as well as in the future, when making assessments of their current health [11].

Studies have examined self-rated health in Asian populations [12,13]. Studies of the quality-of-life of patients [14] and of the general population [15,16] using instruments such as the SF-36 have been conducted in Singapore, but self-rated health has, to our knowledge, hitherto not been described.

Singapore is a multi-ethnic city-state, with a resident population of 3.26 million persons in 2000 [17], of which 76.8% are Chinese, 13.9% Malays and 7.9% Indians. Singapore is wholly urban, and has undergone rapid economic and demographic transitions over the last 40 years, from a poor country with a high fertility rate, to a rich one with one of the lowest fertility rates in Asia and a rapidly aging population. This paper describes self-rated health in Singapore, and examines the socio-economic factors and health behaviours associated with poor health ratings. It concludes by discussing the public health implications of the findings.

## Methods

The dataset was obtained from the National Health Surveillance Survey (NHSS) 2001, which was commissioned by the Ministry of Health, Singapore. The survey methodology has been previously described [18]. NHSS 2001 was a national cross-sectional survey. A 2-stage stratified sampling was performed on a database set containing all dwellings in Singapore, and KISH tables used to identify a respondent from each selected household address. Of 11200 households selected with 9577 identified and eligible residents, 6236 individuals subsequently participated in the survey, giving a response rate of 65.1%. Of these 6236 individuals, information was also obtained from available informants in 1843 persons who were unable to furnish all required information.

The question asking respondents to rate their own health was phrased as follows in English: "In general, how would you rate your health today?" Respondents were given 5 options: very good, good, moderate, bad and very bad to rate their health at the day of interview. We subsequently regrouped their answers into either good (ratings of good or very good) or poor self-rated health (ratings of moderate, bad or very bad) as a dependent variable for logistic regression analyses.

The following socio-demographic factors were studied for possible association with SRH: age (in 5 groups: 18–29 years, 30–39 years, 40–49 years, 50–64 years, 65 years and above), gender, ethnicity (Chinese, Malay, Indian), educational level (in 4 groups: no formal education, up to Primary School Leaving Examination, General Certificate of Education Ordinary level, and General Certificate of Education Advanced level, diploma or degree), monthly average household income (in 4 groups: <S$2000, S$2000-<S$3000, S$3000-<S$5000, S$5000 and above), and marital status (Never married, Married, Separated/Divorced, Widowed)]. Health risk factors were also examined: smoking status (current vs not currently smoking), exercise (those participating in any sports, exercising or walking in the last month vs those who do not), alcohol intake (those who have a drink containing alcohol at least once a week vs those who don't), self-reported body mass index (BMI) {weight (kg)/height (m^2^)} (in 4 groups: BMI<20, BMI 20–25, BMI >25–30, BMI>30)] and self-reports of doctor-diagnosed medical illnesses (those reporting doctor-diagnosed illness vs those who don't), divided into physical and mental illnesses. Mandarin, Malay and Tamil interpreters were used to accommodate respondents who could not speak English.

Data analyses were performed using Stata release 6.0 (Stata Corporation, College Station, Texas 2003). Crude and adjusted odds ratio (OR) and 95% confidence interval (95% CI) were estimated using logistic regression. The analyses were performed using combined data and data stratified by gender. Two-sided tests of significance were used, and significance was set at an alpha level of 0.05. In a sensitivity analysis, the analyses were repeated using the robust estimator of variance to account for possible model mis-specification. The results were very similar. Therefore, only the results using the naïve estimator of variance are presented.

## Results

Table 1 (see Addtional file 1) shows the overall distribution of self-rated health (SRH) responses with regard to socio-economic factors and risk behaviours in the survey population. Table 2 (see Addtional file 2) shows the proportion of all, male, and female respondents reporting very good or good health categorized using a variety of socio-economic factors and risk behaviours Overall, 23.2% of respondents reported their health as moderate, bad or very bad (i.e. poor SRH). Few (less than 0.5%) respondents refused to answer or gave a reply of "Do Not Know". These respondents were excluded from further analyses.

Univariate analyses suggest that gender, ethnicity, marital status, educational level, monthly average household income, age, presence of self-reported doctor-diagnosed illness, current smoking, regular drinking, exercise and BMI were all significantly associated with poor SRH (see Table 3 see Addtional file 3).

In multivariate analysis (Table 3 see Addtional file 3), the odds of reporting poor health were significantly higher in females compared to males (OR = 1.28, 95% CI = 1.08–1.52], among those with a monthly average household income less than S$2000 compared to those with one more than S$5000 a month (1.42, 1.10–1.82), among those reporting at least 1 doctor-diagnosed mental illness compared to persons without doctor-diagnosed illnesses (2.51, 1.64–3.84), and reporting at least 1 doctor-diagnosed physical illness compared to persons without doctor-diagnosed illness (2.83, 2.42–3.31) and current smokers compared to persons who were not currently smoking (1.50, 1.21–1.87). A trend of poor SRH was seen with increasing age; compared to the aged 18–29 years group (test for a linear trend, p = 0.005), the odds of reporting poor health was higher in older age categories (OR = 1.23 in the 30–39 years age-group, 1.39 in the 40–49 age-group, 1.51 in the 50–64 age-group and 3.68 in the 65 and above age-group). Compared to the Chinese, Indians (OR = 0.64,, 0.46–0.90) and Malays (0.70, 0.55–0.88) had lower odds of reporting poor health. Compared to persons with a BMI of 20–25, both those with lower and higher BMIs report poorer SRH: the OR for poor SRH among persons with BMIs less than 20 was 1.28 (95% CI = 1.05–1.56), 1.51 (1.24–1.84) for those with BMI >25 – 30, and 1.90 (1.35–2.67) for those with a BMI >30.

In gender-specific analyses, Indian men reported significantly better SRH than Chinese men; Malay women reported better SRH than Chinese women. The association between household income <S$2000 and poor SRH appeared to be stronger in men (OR = 1.81, 95% CI = 1.25–2.61) than in women (1.15, I = 0.81–1.63). Current smoking was associated with poor SRH in males (1.55, 1.21–1.99) compared to persons who were not currently smoking; this was not seen in females (0.96, 0.56–1.66). Low BMI was associated with poor SRH only in males (OR = 1.51, 95% = 1.09–2.10) and not in females (OR = 1.14, 95% CI = 0.89–1.46).

**Figure 1:**
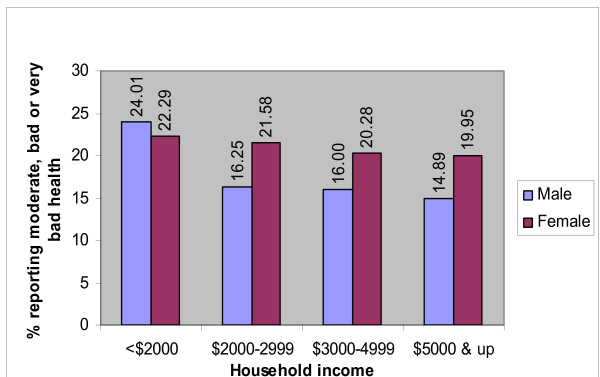
Prevalence of poor self-rated health by gender and household income, adjusted for ethnicity, marital status, educational level, age, self-reported illness, current smoking, regular drinking, exercise and BMI.

Figure 1 shows the predicted prevalence of poor self-rated health by monthly average household income in data stratified by gender and adjusted for marital status, educational level, age, self-reported illness, smoking status, alcohol intake, regular exercise and BMI in the same model. In either gender the prevalence of poor self-rated health is highest in those with average monthly household incomes less than S$2000. The increase in prevalence is more striking in males than in females

**Figure 2:**
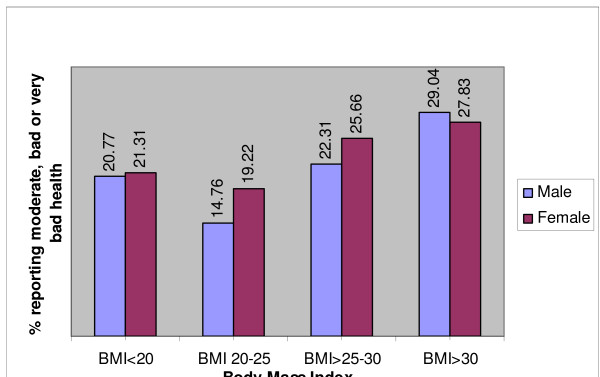
Prevalence of poor self-rated health by gender and BMI, adjusted for ethnicity, marital status, educational level, age, household income, self-reported illness, current smoking, regular drinking, and exercise.

Figure 2 shows the predicted prevalence of poor self-rated health by BMI category in data stratified by gender and adjusted for marital status, educational level, age, self-reported illness, monthly average household income, smoking status, alcohol intake, and regular exercise in the same model. In each gender, the prevalence of poor-self-rated health is lowest in those with a BMI between 20 and 25; however, the relative increments in prevalence in other BMI categories are higher in men than in women.

## Discussion

### General

98.5% of Singaporeans rated their health as very good, good or moderate, with only 1.5% reporting, bad or very bad health (age-adjusted, excluding respondents who refuse to answer or were unable to rate their own health). Vastly different norms have been reported in Eastern Europe, Western Europe and North America. 10.7% of men and 14.4% of women in Estonia reported bad or very bad health. [19] Gilmore *et al *[20] reported 25% of Ukrainian men and 43% of Ukrainian women rated their health as poor or very poor. A 1996 study [21] presented data showing that 16% of the adult population in Rotterdam reported their health as not very healthy or not healthy at all (the lowest 2 of 5 ordinal categories), and Zack *et al *[22] reported that 15.5% of adult Americans rated their health as fair or poor (the lowest 2 of 5 ordinal categories) in 2001. Other Chinese populations also appear to report poorer self-rated health [23,24]. In a 1998 study in Shanghai of the elderly (65 years and up), 50.6% of respondents rated their health in the lowest 2 categories on a 4-category scale. (Yu et al 1998) [23]. In our study 7.0% rated theirs in the lowest 2 of a 5-category scale. Inter-country comparisons should be undertaken with care. There are significant differences in methodology between countries, for example the kinds of rating scale used, the method involved in eliciting a response, and the way in which the question was phrased. However, our impression is that Singaporeans in general appear more positive in their ratings of health compared to many other countries.

### Age

Age and self-reported illnesses were significantly and strongly associated with poor self-reported health (their adjusted odds ratios ≥ 3). This has been consistently shown in multiple studies [6,20,25] and it suggests that people assess their current well-being and illnesses together with other intangible factors such as their expectation of future health in rating their current health.

### Gender

Gender differences in self ratings of health have not been consistent. Women were more likely to report poor self-rated health than men in our study, and a study in Estonia reported that women had higher ratings for health, after controlling for physical health status, emotional distress and locus of control. [19] On the other hand, a comparison of 2 populations in Karelia, 1 Russian and the other Finn, showed that Russian women were less likely than Russian men to report their health as quite good or very good, whereas the Finn women were more likely than Finn men to do so [26]. A Pakistan study also reported poorer ratings in women than men [12]. This suggests that cultural factors in different ethnicities may play a role.

Women living in transitional or rapidly changing societies may experience unique stresses as a result of trying to accommodate new role expectations. The social status of women may also be important: A US study reported that women living in states where the status of women is lower were more likely to report poor health [27]. Depression and anxiety disorders are more common in women [28], and these conditions may impact the ways people evaluate their health status.

### Ethnicity

Previous studies have also shown that poor SRH is independently associated with ethnicity in multiethnic communities [29,30]. It is unusual that minority groups report better SRH overall. Ethnic differences may represent residual confounding by socio-economic influences that have not been adequately accounted for. (This is unlikely to account fully for the differences seen, since income and education levels of Malays and Indians are not higher than those seen in the Chinese [17].) Other studies conducted in Singapore looking at quality of life have also reported ethnic differences [14-16]. There appears to be no consistent direction in these differences: the diabetes study [14] reported that Indian diabetics were most likely to report impaired quality of life; in the study on healthy adolescents [16], Indians fared best on overall quality of life. Normative ratings may differ between different ethnic groups, reflecting differing cultural norms.

Self-rated health has been associated with social trust [31]and social networks [32]. Malay and Indian communities may possess better social networks and experience greater social trust as a result of stronger family and religious ties, and this may have been reflected in better SRH. The questionnaire was not translated into other languages; instead interpreters who were free to translate as appropriate were used for respondents who did not know English. As such, differences in phrasing used by different interpreters may also have been a factor.

### Education

Previous studies have shown inverse relationships between educational level (after adjusting for material deprivation) and self-rated health [33]. In our study, this relationship disappeared after adjustment for household income. Expressing education in terms of years of education instead of level of attainment did not show a significant association either (data not shown). The relationship between educational status and self-rated health may lie in the self-perceived efficacy in safeguarding one's health that education confers; in a small, highly urbanized society like Singapore, perceptions of efficacy may not differ much. Health education and promotion messages in Singapore may be delivered in avenues that do not require significant literacy (for example, radio and television programmes) and hence poorly educated persons do not perceive a significant disadvantage. The effect of education may be partially mediated through the impact of household income. Excluding the household income variable from the multivariate model showed that those with no formal education had an OR of 1.29 (95% CI = 1.00–1.66) of reporting poor SRH compared to those with the highest (A-levels, diploma or degree) level of education attainment.

### Health behaviours and BMI

Previous studies have variously reported that alcohol use, lack of exercise and being a current smoker were associated with poorer SRH [23,34,35]. A Japanese study reported a positive association between moderate alcohol use and good SRH [36]. However, our categories for exercise are crude and may have failed to capture the true effects of consistent, sustained exercise on self-rated health. Few of our respondents drank alcohol, and this may have contributed to the lack of power in the multivariate analyses.

Smoking results in symptoms such as poor effort tolerance, chronic cough and hoarse voice, which respondents may interpret as poor health. Respondents who are distressed, sad or worried are more likely to report poor self-rated health and these persons are also more likely to smoke (data not shown), suggesting that the relationship between smoking and poor SRH may be confounded. However, this relationship persists after adjustment for self-reported pain, mobility, and distress, sadness, and worry, (results not shown) suggesting that the first interpretation is more likely.

Obesity and underweight have been associated previously with poor SRH [37]. This association may reflect "symptoms" such as poor effort tolerance. Overweight and obese persons could also be considering their likely future health trajectory in their health evaluations, as the medical problems associated with obesity are well-known.

### Gender differences in the effect of various factors on SRH

Other researchers have not shown gender differences in the relationship between household income and self-rated health. Leinsalu [19] reported that the inverse association was stronger in women than men in his data. A reason for our contrary findings (with association between low household income and poor SRH only in men) could be that the relationship seen in men is confounded by occupation, a factor that we did not include in our analyses and which has been shown to be important in explaining the relationship between income and health in men [33].

Male current smokers in our study smoked more on average than female current smokers (15.0 vs. 12.4 cigarettes a day) and as a result may suffer more from the effects of smoking. Female smokers may be less aware of the potential consequences of long-term smoking. Men and women may be smoking for different reasons: Men may be using it to cope with stress and anxiety, and women, for the "thrill" it provides. However, adjusting for self-rated pain or discomfort, difficulty moving around, and distress, sadness and worry over the 30 days preceding the interview slightly attenuated (to 1.43) but did not obliterate the association in men.

The different relationships between BMI and poor SRH in men and women were unusual. We had speculated that self- ratings of health were associated with body image, and so we hypothesized that while men would be more likely to associate being underweight with poor SRH then women, we also expected that the association between high BMI and poor SRH would be stronger in women than in men because of the stronger societal pressures exerted on women with regard to obesity. We observed a stronger association between high BMI and poor self-rated health in men than in women. This suggests that our initial hypothesis is wrong, and people do not relate health with body image. It is difficult to explain why high BMIs might be associated with poorer SRH in men than in women; our speculation is that men may compensate more poorly in obesity and experience more symptoms related to obesity such as shortness of breath.

### Limitations

Our study design is cross-sectional in nature and it is hence difficult to establish cause-effect relationships between self-rated health and the various socio-economic factors and health behaviours. A longitudinal study is needed to ascertain these relationships in future. This study however, sampled a representative cross-section of Singaporean society and has a fairly large sample size.

Other limitations are that our sampling took into account only non-institutionalised individuals, and excluded persons living long-term in nursing homes, hospitals for the chronic sick, and those in correctional institutes. Such a design may biase our measurement of self-rated health towards the positive end. Informants were used in proxy interviews to obtain some information (including, in some cases, ratings of health) in up to 30% of respondents. These proxy interviews could introduce error in our ascertainment of self-rated health. However, there was no significant association between whether proxy interviews were used and poor self-rated health. (p value = 0.15.)

We also did not perform standardized translations of the questions in the other official languages (Malay, Mandarin and Tamil) for the interviewers. Interviewers were hence free to translate the questions in whichever manner they deemed appropriate. This could have affected the measurement and interpretation of self-rated health, in particular in the comparisons between racial groups.

We believe that the same relationship between poor self-rated health and increased mortality that has been observed worldwide is present in Singapore and this relationship should be confirmed. Unfortunately, we are unable to extend our study, as the removal of identifiers has meant that we cannot track the mortality experience of our study population. We are also unable to take serial measurements of self-rated health, which may confer more information than a single point measurement as we have done.

### Public Health Implications

While the cause-effect direction of the inverse income-SRH relationship cannot be ascertained in a cross-sectional survey, numerous studies have documented similar inverse relationships between personal and family income and income inequality, and SRH [38-40] and it is believed that the difference in SRH is significant and reflects inequalities in health outcomes in different socio-economic groups. It is important to monitor this relationship through regular national longitudinal surveys. Widening of the self-rated health gap between people in different income groups may alert authorities to a growing problem, and solutions such as increasing health services and health promotional activities for the poor can be implemented.

The relationship between smoking and poor self-rated health could be used to bolster Singapore's anti-smoking campaign. While the long-term effects of smoking are well-known, such effects may not be taken into sufficient account by the young. A message emphasising that smokers feel less healthy may resonate better with the young. The gender difference in the association between smoking and poor SRH should be further studied to determine the reasons for this difference.

The associations between SRH and BMI may also be useful in anti-obesity campaigns; highlighting that persons with healthy BMIs report better health may be a useful adjunct to persuade people to maintain healthy weights.

In summary, gender, ethnicity, household income, age, self-reported illness, current smoking and BMI were significantly associated with poor self-rated health even after the adjustment. There were gender differences in the association of household income, smoking status and BMI to self-rated health.

## Competing interests

The author(s) declare that they have no competing interests.

## Authors' contributions

LWY drafted manuscript and performed statistical analysis

SM provided statistical advice and support

DH, VB and CSK designed the community health survey upon which this study was based, and offered advice regarding the analysis and manuscript

## Pre-publication history

The pre-publication history for this paper can be accessed here:



## Supplementary Material

Additional file 1 Quality of life among Singaporeans. Click here for file

Additional file 2 Proportion of respondents reporting very good or good health, by various socio-demographic and health behaviour factors. Click here for file

Additional file 3 Unadjusted and adjusted odds ratio (OR) and 95% confidence intervals (95% CI) of reporting moderate, bad or very bad health by socio-economic factors and health risk factors. Click here for file
