# Supplementary material for: Gender, ethnicity, health behaviour & self-rated health in Singapore
Source: BMC Public Health. 2007 Jul 27;7:184. doi: 10.1186/1471-2458-7-184 (PMC1976324; doi:10.1186/1471-2458-7-184)
Supplement: Additional file 1 — Quality of life among Singaporeans. [file 1471-2458-7-184-S1.doc]

**Table 1: Quality of life among Singaporeans**

| **Self-rated health** | **Very good** | **Good** | **Moderate** | **Fair/**  **poor** | **Refuse** | **Don’t know** | **Total** |
| --- | --- | --- | --- | --- | --- | --- | --- |
| ***Overall*** | 1398  (22.4) | 3360  (53.9) | 1365  (21.9) | 79  (1.3) | 1  (0.0) | 33  (0.5) | 6236  (100) |
| ***Gender*** |  |  |  |  |  |  |  |
| Male | 774  (25.0) | 1651  (53.3) | 617  (19.9) | 37  (1.2) | 1  (0) | 19  (0.6) | 3099  (100) |
| Female | 624 (19.9) | 1709  (54.5) | 748  (23.8) | 42  (1.3) | 0  (0) | 14  (0.5) | 3137  (100) |
| ***Ethnicity*** |  |  |  |  |  |  |  |
| Chinese | 1102  (22.5) | 2602  (53.2) | 1092  (22.3) | 68  (1.4) | 1  (0) | 27  (0.6) | 4892  (100) |
| Indian | 112  (28.5) | 201  (51.2) | 74  (18.8) | 4  (1.0) | 0  (0) | 2  (0) | 393  (100) |
| Malay | 161  (18.9) | 512  (60.2) | 171  (20.1) | 4  (0.5) | 0  (0) | 3  (0.4) | 851  (100) |
| Others | 23  (23.0) | 45  (45.0) | 28  (28.0) | 3  (3.0) | 0  (0) | 1  (1) | 100  (100) |
| ***Marital status*** |  |  |  |  |  |  |  |
| Never married | 480  (31.6) | 755  (49.6) | 264  (17.4) | 17  (1.1) | 0  (0) | 6  (0.4) | 1522  (100) |
| Married | 866  (20.2) | 2423  (56.4) | 944  (22.0) | 39  (0.9) | 1  (0) | 25  (0.6) | 4298  (100) |
| Separated/  Divorced | 29  (17.9) | 85  (52.5) | 44  (27.2) | 3  (1.9) | 0  (0) | 1  (0.6) | 162  (100) |
| Widowed | 23  (9.2) | 96  (38.3) | 111  (44.2) | 20  (8.0) | 0  (0) | 1  (0.4) | 251  (100) |
| ***Education*** |  |  |  |  |  |  |  |
| No formal | 100  (12.7) | 368  (46.6) | 283  (35.9) | 36  (4.6) | 0  (0) | 2  (0.3) | 789  (100) |
| PSLE | 259  (19.1) | 739  (54.4) | 338  (24.9) | 16  (1.2) | 1  (0.1) | 6  (0.4) | 1359  (100) |
| O-level | 518  (24.7) | 1154  (55.1) | 399  (19.1) | 7  (0.3) | 0  (0) | 16  (0.8) | 2094  (100) |
| A-level/ Dip/ Degree | 520  (26.1) | 1097  (55.1) | 345  (17.3) | 20  (1.0) | 0  (0) | 4  (0.2) | 1991  (100) |
| ***Household income (S$)*** |  |  |  |  |  |  |  |
| <2000 | 301  (21.3) | 712  (50.4) | 362  (25.6) | 27  (1.9) | 0  (0) | 11  (0.1) | 1413  (100) |
| 2001-<3000 | 294  (24.0) | 681  (55.5) | 237  (19.3) | 7  (0.1) | 1  (0) | 7  (0.1) | 1227  (100) |
| 3001-<5000 | 289  (23.3) | 701  (56.6) | 240  (19.4) | 6  (0.5) | 0  (0) | 3  (0.2) | 1239  (100) |
| >5000 | 246  (22.8) | 622  (57.7) | 198  (18.4) | 5  (0.5) | 0  (0) | 7  (0.6) | 1078  (100) |
| ***Age*** |  |  |  |  |  |  |  |
| 18-29 | 453  (33.0) | 703  (51.2) | 199  (14.5) | 11  (0.8) | 0  (0) | 6  (0.4) | 1372  (100) |
| 30-39 | 367  (21.9) | 993  (59.2) | 304  (18.1) | 3  (0.2) | 0  (0) | 9  (0.5) | 1676  (100) |
| 40-49 | 354  (20.8) | 965  (56.7) | 356  (20.9) | 18  (1.1) | 1  (0) | 9  (0.5) | 1703  (100) |
| 50-64 | 184  (16.9) | 552  (50.8) | 325  (29.9) | 19  (1.7) | 0  (0) | 7  (0.6) | 1087  (100) |
| 65 and above | 40  (10.1) | 147  (36.9) | 181  (45.5) | 28  (7.0) | 0  (0) | 2  (0.5) | 398  (100) |
| ***Self-reported mental illness^1^*** |  |  |  |  |  |  |  |
| No | 1383  (22.7) | 3311  (54.3) | 1302  (21.4) | 68  (1.1) | 1  (0) | 32  (0.5) | 6097  (100) |
| Yes | 15  (10.8) | 49  (35.3) | 63  (45.3) | 11  (7.9) | 0  (0) | 1  (0.7) | 139  (100) |
| ***Self-reported physical illness^2^*** |  |  |  |  |  |  |  |
| No | 1065  (25.9) | 2401  (58.4) | 604  (14.7) | 17  (0.4) | 1  (0) | 23  (0.6) | 4111  (100) |
| Yes | 333  (15.7) | 959  (45.1) | 761  (35.8) | 57  (2.7) | 4  (0.2) | 11  (0.5) | 2125  (100) |
| ***Current smoking^3^*** |  |  |  |  |  |  |  |
| No | 1199  (22.7) | 2853  (54.1) | 1129  (21.4) | 64  (1.2) | 1  (0) | 29  (0.5) | 5275  (100) |
| Yes | 199  (20.7) | 507  (52.8) | 236  (24.6) | 15  (1.6) | 0  (0) | 4  (0.4) | 961  (100) |
| ***Regular drinking^4^*** |  |  |  |  |  |  |  |
| No | 1347  (22.7) | 3204  (53.9) | 1280  (21.5) | 77  (1.3) | 1  (0) | 32  (0.5) | 5941  (100) |
| Yes | 51  (17.3) | 156  (52.9) | 85  (28.8) | 2  (0.7) | 0  (0) | 1  (0.3) | 295  (100) |
| ***Exercise^5^*** |  |  |  |  |  |  |  |
| No | 713  (20.9) | 1838  (53.9) | 786  (23.1) | 56  (1.6) | 1  (0) | 14  (0.4) | 3408  (100) |
| Yes | 685  (24.2) | 1522  (53.8) | 579  (20.5) | 23  (0.8) | 0  (0) | 19  (0.7) | 2828  (100) |
| ***Body Mass Index^6^*** |  |  |  |  |  |  |  |
| < 20 | 327  (25.1) | 682  (52.3) | 278  (21.3) | 12  (0.9) | 0  (0) | 6  (0.5) | 1305  (100) |
| 20-25 | 693  (23.7) | 1644  (56.1) | 545  (18.6) | 33  (1.1) | 0  (0) | 15  (0.5) | 2930  (100) |
| >25 to 30 | 187  (18.4) | 540  (53.2) | 269  (26.5) | 12  (1.2) | 1  (0.1) | 6  (0.6) | 1015  (100) |
| >30 | 44  (18.3) | 113  (46.9) | 79  (32.8) | 3  (1.2) | 0  (0) | 2  (0.8) | 241  (100) |

*^1^ Self-reports of doctor-diagnosed depression, anxiety or sleep problems*

*^2^ Self-reports of doctor-diagnosed diabetes mellitus, hypertension, arthritis, heart disease, chronic bronchitis and emphysema, allergic respiratory disease and asthma, back pain and disc problems, migraine, stroke, , hearing and vision problems, gastritis and stomach ulcer, tumours and cancer, high cholesterol, chronic renal disease or other self-reports of doctor-diagnosed illnesses.*

*^3^ Current smokers are defined as those who report daily or occasional smoking and have ever smoked at least 100 cigarettes (lifetime).*

*^4^ Regular drinkers are defined as those who have a drink containing alcohol at least once a week.*

*^5^Exercise are persons who report participating in any sports, exercise or walking in the past month*

*^6^BMI equals self reported weight in kilogrammes divided by the square of self-reported height, in metres*
