# Supplementary material for: Gender, ethnicity, health behaviour & self-rated health in Singapore
Source: BMC Public Health. 2007 Jul 27;7:184. doi: 10.1186/1471-2458-7-184 (PMC1976324; doi:10.1186/1471-2458-7-184)
Supplement: Additional file 2 — Proportion of respondents reporting very good or good health, by various socio-demographic and health behaviour factors. [file 1471-2458-7-184-S2.doc]

**Table 2 Proportion of respondents reporting very good or good health, by various socio-demographic and health behaviour factors**

|  | **Males** | | | | **Females** | | | | **Combined** | | | |
| --- | --- | --- | --- | --- | --- | --- | --- | --- | --- | --- | --- | --- |
| Variable | Self-Rated Health:  Very good/Good  (%) | | Total | | Self-Rated Health:  Very good/Good  (%) | | Total | | Self-Rated Health:  Very good/Good  (%) | | Total | |
| **Overall** | 2425 (78.8) | | 3079 | | 2333 (74.7) | | 3123 | | 4758 (76.3) | | 6236 | |
|  |  | |  | |  | |  | |  | |  | |
| **Gender** |  | |  | |  | |  | |  | |  | |
| Male | - | | - | | - | | - | | 2425 (78.3) | | 3099 | |
| Female | - | | - | | - | | - | | 2333 (74.4) | | 3137 | |
| **Age group (years)** |  | |  | |  | |  | |  | |  | |
| 18-29 | 571 (87.6) | | 652 | | 585 (81.9) | | 714 | | 1156 (84.3) | | 1372 | |
| 30-39 | 690 (83.5) | | 826 | | 670 (79.7) | | 841 | | 1360 (81.2) | | 1676 | |
| 40-49 | 670 (77.4) | | 866 | | 649 (78.5) | | 827 | | 1319 (77.4) | | 1703 | |
| 50-64 | 389 (71.2) | | 546 | | 347 (65.0) | | 534 | | 736 (67.7) | | 1087 | |
| 65 and above | 105 (55.6) | | 189 | | 82 (39.6) | | 207 | | 187 (47) | | 398 | |
| **Ethnic group** |  | |  | |  | |  | |  | |  | |
| Chinese | 1888 (78.5) | | 2405 | | 1818 (73.9) | | 2459 | | 3704 (75.7) | | 4892 | |
| Indian | 161 (83.0) | | 194 | | 152 (77.2) | | 197 | | 313 (79.6) | | 393 | |
| Malay | 338 (79.5) | | 426 | | 335 (79.4) | | 422 | | 673 (79.1) | | 851 | |
| Others | 40 (74.1) | | 54 | | 28 (62.2) | | 45 | | 68 (68) | | 100 | |
| **Marital status** |  | |  | |  | |  | |  | |  | |
| Never married | 698 (82.6) | | 845 | | 537 (80.0) | | 671 | | 1235 (81.1) | | 1522 | |
| Married | 1656( 78.0) | | 2124 | | 1633 (76.0) | | 2148 | | 3289 (76.5) | | 4298 | |
| Separated/Divorced | 45 (76.3) | | 59 | | 69 (67.6) | | 102 | | 114 (70.4) | | 162 | |
| Widowed | 26 (52.0) | | 50 | | 93 (46.5) | | 200 | | 119 (47.4) | | 251 | |
| **Educational level** |  | |  | |  | |  | |  | |  | |
| No formal | 183 (61.6) | | 297 | | 285 (58.2) | | 490 | | 468 (59.3) | | 789 | |
| PSLE | 512 (74.2) | | 690 | | 486 (73.4) | | 662 | | 998 (73.4) | | 1359 | |
| O-level | 847 (81.9) | | 1034 | | 825 (79.0) | | 1044 | | 1672 (79.8) | | 2094 | |
| A-level/ Dip/ Degree | 882 (83.4) | | 1057 | | 735 (79.5) | | 925 | | 1617 (81.2) | | 1991 | |
| **Household income (S$)** | |  | |  | |  | |  | |  | |  |
| <2000 | 489 (71.8) | | 681 | | 524 (72.7) | | 721 | | 1013 (71.7) | | 1413 | |
| 2000-<3000 | 525( 82.4) | | 637 | | 450 (77.3) | | 582 | | 975 (79.5) | | 1227 | |
| 3000-<5000 | 491 (82.4) | | 596 | | 499 (78.0) | | 640 | | 990 (79.9) | | 1239 | |
| 5000 and above | 464 (83.2) | | 559 | | 404 (78.9) | | 512 | | 868 (80.5) | | 1078 | |
| **Self-reported mental illness^1^** |  | |  | |  | |  | |  | |  | |
| No | 2392 (79.1) | | 3023 | | 2302 (75.7) | | 3041 | | 4694 (77.4) | | 6064 | |
| Yes | 33 (58.9) | | 56 | | 31 (37.8) | | 82 | | 64 (46.4) | | 138 | |
| **Self reported physical illness^2^** |  | |  | |  | |  | |  | |  | |
| No | 1788 (85.8) | | 2085 | | 1678 (83.8) | | 2003 | | 3466 (84.8) | | 4088 | |
| Yes | 637 (64.1) | | 994 | | 655 (58.5) | | 1120 | | 1292 (61.1) | | 2114 | |
| **Current smoking^3^** |  | |  | |  | |  | |  | |  | |
| No | 1818 (80.5) | | 2258 | | 2234 (74.8) | | 2987 | | 4052 (76.8) | | 5275 | |
| Yes | 607 (73.9) | | 821 | | 99 (72.8) | | 136 | | 706 (73.5) | | 961 | |
| **Regular drinking^4^** |  | |  | |  | |  | |  | |  | |
| No | 2250 (79.5) | | 2830 | | 2301 (74.8) | | 3078 | | 4551 (76.6) | | 5941 | |
| Yes | 175 (70.3) | | 249 | | 32 (71.1) | | 45 | | 207 (70.2) | | 295 | |
| **Exercise^5^** |  | |  | |  | |  | |  | |  | |
| No | 1188 (77.0) | | 1543 | | 1363 (73.7) | | 1850 | | 2551 (74.9) | | 3408 | |
| Yes | 1237 (80.5) | | 1536 | | 970 (76.2) | | 1273 | | 2207 (78.0) | | 2828 | |
| **Body Mass Index^6^** |  | |  | |  | |  | |  | |  | |
| < 20 | 311 (78.9) | | 394 | | 698 (77.1) | | 905 | | 1009 (77.3) | | 1305 | |
| 20-25 | 1310 (82.5) | | 1588 | | 1027 (77.4) | | 1327 | | 2337 (79.8) | | 2930 | |
| >25 to 30 | 461 (74.0) | | 623 | | 266 (69.1) | | 385 | | 727 (71.6) | | 1015 | |
| >30 | 81 (66.4) | | 122 | | 76 (65.0) | | 117 | | 157 (65.2) | | 241 | |

*^1^ Self-reports of doctor-diagnosed depression, anxiety or sleep problems*

*^2^ Self-reports of doctor-diagnosed diabetes mellitus, hypertension, arthritis, heart disease, chronic bronchitis and emphysema, allergic respiratory disease and asthma, back pain and disc problems, migraine, stroke, , hearing and vision problems, gastritis and stomach ulcer, tumours and cancer, high cholesterol, chronic renal disease or other self-reports of doctor-diagnosed illnesses.*

*^3^ Current smokers are defined as those who report daily or occasional smoking and have ever smoked at least 100 cigarettes (lifetime).*

*^4^ Regular drinkers are defined as those who have a drink containing alcohol at least once a week.*

*^5^Exercise are persons who report participating in any sports, exercise or walking in the past month*

*^6^BMI equals self reported weight in kilogrammes divided by the square of self-reported height, in metres*
