# Supplementary material for: Gender, ethnicity, health behaviour & self-rated health in Singapore
Source: BMC Public Health. 2007 Jul 27;7:184. doi: 10.1186/1471-2458-7-184 (PMC1976324; doi:10.1186/1471-2458-7-184)
Supplement: Additional file 3 — Unadjusted and adjusted odds ratio (OR) and 95% confidence intervals (95% CI) of reporting moderate, bad or very bad health by socio-economic factors and health risk factors. [file 1471-2458-7-184-S3.doc]

**Table 3 Unadjusted and adjusted^1^ odds ratios (OR) and 95% confidence intervals (95% CI) of reporting moderate, bad or very bad health by socio-economic** factors and health risk factors

|  | Male | |  | Female | |  | Combined | |
| --- | --- | --- | --- | --- | --- | --- | --- | --- |
| Variable | Unadjusted  OR (95% CI) | Adjusted  OR (95% CI) |  | Unadjusted  OR (95% CI) | Adjusted  OR (95% CI) |  | Unadjusted  OR (95% CI) | Adjusted  OR (95% CI) |
| **Gender** |  |  |  |  |  |  |  |  |
| Male | - | - |  | - | - |  | 1.00 | 1.00 |
| Female | - | - |  | - | - |  | 1.26 (1.12-1.41) | 1.28 (1.08-1.52) |
| **Age group (years)** |  |  |  |  |  |  |  |  |
| 18-29 | 1.00 | 1.00 |  | 1.00 | 1.00 |  | 1.00 | 1.00 |
| 30-39 | 1.39 (1.03-1.87) | 1.28 (0.87-1.89) |  | 1.16 (0.90-1.49) | 1.23 (0.87-1.74) |  | 1.24 (1.03-1.51) | 1.23 (0.96-1.59) |
| 40-49 | 2.06 (1.56-2.73) | 1.64 (1.09-2.48) |  | 1.24 (0.97-1.60) | 1.18 (0.83-1.70) |  | 1.56 (1.30-1.88) | 1.39 (1.06-1.81) |
| 50-64 | 2.85 (2.11-3.83) | 1.67 (1.06-2.63) |  | 2.44 (1.88-3.17) | 1.41 (0.93-2.15) |  | 2.57 (2.12-3.13) | 1.51 (1.12-2.05) |
| 65 and above | 5.64 (3.90-8.16) | 3.25 (1.65-6.39) |  | 6.91 (4.93-9.69) | 4.55 (2.29-9.04) |  | 6.15 (4.81-7.87) | 3.68 (2.30-5.89) |
| **Ethnic group** |  |  |  |  |  |  |  |  |
| Chinese | 1.00 | 1.00 |  | 1.00 | 1.00 |  | 1.00 | 1.00 |
| Malay | 0.95 (0.73-1.22) | 0.85 (0.62-1.18) |  | 0.74 (0.57-0.95) | 0.55 (0.39-0.77) |  | 0.83 (0.69-0.99) | 0.70 (0.55-0.88) |
| Indian | 0.75 (0.51-1.10) | 0.51 (0.31-0.85) |  | 0.84 (0.59-1.19) | 0.77 (0.50-1.20) |  | 0.80 (0.62-1.03) | 0.64 (0.46-0.90) |
| Others | 1.27 (0.69-2.36) | 0.86 (0.38-1.95) |  | 1.72 (0.94-3.17) | 1.33 (0.60-2.93) |  | 1.46 (0.95-2.24) | 1.10 (0.63-1.91) |
| **Marital status** |  |  |  |  |  |  |  |  |
| Never married | 1.00 | 1.00 |  | 1.00 | 1.00 |  | 1.00 | 1.00 |
| Married | 1.34 (1.09-1.65) | 0.85 (0.62-1.17) |  | 1.26 (1.02-1.56) | 1.03 (0.76-1.41) |  | 1.31 (1.13-1.52) | 0.94 (0.75-1.16) |
| Separated/Divorced | 1.48 (0.79-2.76) | 0.75 (0.32-1.76) |  | 1.92 (1.21-3.02) | 1.47 (0.81-2.67) |  | 1.81 (1.26-2.61) | 1.08 (0.67-1.74) |
| Widowed | 4.38 (2.45-7.85) | 2.11 (0.70-6.32) |  | 4.61 (3.29-6.45) | 1.43 (0.77-2.63) |  | 4.84 (3.66-6.40) | 1.54 (0.93-2.55) |
| **Educational level** |  |  |  |  |  |  |  |  |
| No formal | 3.14 (2.36-4.17) | 1.03 (0.63-1.69) |  | 2.78 (2.19-3.54) | 1.00 (0.64-1.54) |  | 3.02 (2.52-3.62) | 1.05 (0.76-1.44) |
| PSLE | 1.75 (1.38-2.22) | 0.78 (0.54-1.12) |  | 1.40 (1.11-1.77) | 0.88 (0.62-1.27) |  | 1.57 (1.33-1.86) | 0.87 (0.68-1.12) |
| O-level | 1.11 (0.89-1.40) | 0.92 (0.68-1.23) |  | 1.03 (0.83-1.28) | 0.82 (0.62-1.10) |  | 1.08 (0.92-1.26) | 0.89 (0.73-1.10) |
| A-level/ Dip/ Degree | 1.00 | 1.00 |  | 1.00 | 1.00 |  | 1.00 | 1.00 |
| **Household income (S$)** |  |  |  |  |  |  |  |  |
| <2000 | 1.92 (1.45-2.53) | 1.81 (1.25-2.61) |  | 1.41 (1.08-1.84) | 1.15 (0.81-1.63) |  | 1.64 (1.35-1.99) | 1.42 (1.10-1.82) |
| 2000-<3000 | 1.04 (0.77-1.41) | 1.11 (0.77-1.59) |  | 1.10 (0.82-1.46) | 1.10 (0.79-1.55) |  | 1.07 (0.87-1.32) | 1.09 (0.85-1.40) |
| 3000-<5000 | 1.04 (0.77-1.41) | 1.09 (0.78-1.53) |  | 1.06 (0.80-1.40) | 1.02 (0.74-1.40) |  | 1.06 (0.86-1.31) | 1.05 (0.83-1.32) |
| 5000 and above | 1.00 | 1.00 |  | 1.00 | 1.00 |  | 1.00 | 1.00 |
| **Self-reported mental illness^2^** |  |  |  |  |  |  |  |  |
| No | 1.00 | 1.00 |  | 1.00 | 1.00 |  | 1.00 | 1.00 |
| Yes | 2.64 (1.54-4.53) | 1.97 (1.02-3.82) |  | 5.12 (3.25-8.07) | 3.11 (1.76-5.50) |  | 3.96  (2.82-5.57) | 2.51 (1.64-3.84) |
| **Self-reported physical illness^3^** |  |  |  |  |  |  |  |  |
| No | 1.00 | 1.00 |  | 1.00 | 1.00 |  | 1.00 | 1.00 |
| Yes | 3.37 (2.82-4.03) | 2.75 (2.19-3.45) |  | 3.67 (3.10-4.34) | 3.05 (2.45-3.80) |  | 3.55 (3.14-4.01) | 2.83 (2.42-3.31) |
| **Current smoking^4^** |  |  |  |  |  |  |  |  |
| No | 1.00 | 1.00 |  | 1.00 | 1.00 |  | 1.00 | 1.00 |
| Yes | 1.46 (1.21-1.76) | 1.55 (1.21-1.99) |  | 1.11 (0.75-1.63) | 0.96 (0.56-1.66) |  | 1.21 (1.03-1.41) | 1.50 (1.21-1.87) |
| **Regular drinking^5^** |  |  |  |  |  |  |  |  |
| No | 1.00 | 1.00 |  | 1.00 | 1.00 |  | 1.00 | 1.00 |
| Yes | 1.64 (1.23-2.19) | 1.44 (0.99-2.08) |  | 1.20 (0.63-2.30) | 1.42 (0.60-3.37) |  | 1.41 (1.09-1.82) | 1.34 (0.96-1.87) |
| **Exercise^6^** |  |  |  |  |  |  |  |  |
| No | 1.00 | 1.00 |  | 1.00 | 1.00 |  | 1.00 | 1.00 |
| Yes | 0.81 (0.68-0.96) | 0.93 (0.74-1.17) |  | 0.87 (0.74-1.03) | 0.84 (0.68-1.05) |  | 0.83 (0.73-0.93) | 0.88 (0.75-1.03) |
| **Body Mass Index^7^** |  |  |  |  |  |  |  |  |
| <20 | 1.26 (0.96-1.65) | 1.51 (1.09-2.10) |  | 1.02 (0.83-1.24) | 1.14 (0.89-1.46) |  | 1.16 (0.99-1.36) | 1.28 (1.05-1.56) |
| 20-25 | 1.00 | 1.00 |  | 1.00 | 1.00 |  | 1.00 | 1.00 |
| >25-30 | 1.66 (1.33-2.07) | 1.66 (1.28-2.15) |  | 1.53 (1.19-1.97) | 1.45 (1.06-1.98) |  | 1.56 (1.33-1.84) | 1.51 (1.24-1.84) |
| >30 | 2.39 (1.60-3.55) | 2.36 (1.50-3.73) |  | 1.85 (1.24-2.76) | 1.62 (0.97-2.70) |  | 2.11 (1.59-2.80) | 1.90 (1.35-2.67) |

*^1^ Multivariate logistic regression, adjusting for the other factors shown in the table*

*^2^ Self-reports of doctor-diagnosed depression, anxiety or sleep problems*

*^3^ Self-reports of doctor-diagnosed diabetes mellitus, hypertension, arthritis, heart disease, chronic bronchitis and emphysema, allergic respiratory disease and asthma, back pain and disc problems, migraine, stroke, , hearing and vision problems, gastritis and stomach ulcer, tumours and cancer, high cholesterol, chronic renal disease or other self-reports of doctor-diagnosed illnesses.*

*^4^ Current smokers are defined as those who report daily or occasional smoking and have ever smoked at least 100 cigarettes (lifetime).*

*^5^ Regular drinkers are defined as those who have a drink containing alcohol at least once a week.*

*^6^Exercise are persons who report participating in any sports, exercise or walking in the past month*

*^7^BMI equals self-reported weight in kilogrammes divided by the square of self-reported height, in metres*
